# The Tensile Behavior of Hybrid Bonded Bolted Composite Joints: 3D-Digital Image Correlation versus Finite Element Analysis

**DOI:** 10.3390/ma17071675

**Published:** 2024-04-05

**Authors:** Raphael Blier, Leila Monajati, Masoud Mehrabian, Rachid Boukhili

**Affiliations:** 1 Department of Mechanical Engineering, Royal Military College of Canada, Kingston, ON K7K 7B4, Canada; raphblier@gmail.com; 2 Department of Mechanical Engineering, Polytechnique, Montreal, QC H3T 1J4, Canada; leila.monajati@polymtl.ca (L.M.); masoud.mehrabian86@gmail.com (M.M.)

**Keywords:** finite element analysis, composite materials, hybrid bolted/bonded joints, DIC experimental results

## Abstract

This study examines the behavior of hybrid bolted/bonded (HBB) joints loaded in tensile shear comprising plain weave carbon/epoxy laminates in quasi-isotropic (QI) and cross-ply (CP) layups. It proposes a combined approach of 3D digital image correlation and finite element analysis (FEA) to assess their behavior. To apply the FEA simulation accurately, a single layer of plain fabric was replaced with ${\left[0/90\right]}_{s}$ lamination. Experimental standard open-hole tension test results, as well as only bolted (OB) and HBB, along with FEA predictions, confirmed the accuracy of the substitution method. The FEA, calibrated by experimental results, provides insight into the distinctive characteristics of HBB joints in comparison with bonded and bolted joints. Critical considerations include material properties, damage modeling, adhesive characteristics, and mass scaling. The FEA results underscored the pivotal role of adhesives in HBB joints, rendering them akin solely to bonded configurations. HBB joints retain their geometry better than OB joints with considerably less out-of-plane displacement, following a sinusoidal trend. Moreover, the overall behavior of the two layups demonstrates that CP benefits from having higher strength than QI, especially at the critical hole located closer to the grip side.

## 1. Introduction

Hybrid bolted/bonded (HBB) composite joints are an interesting alternative to bolted or bonded composite joints for the reduction of peel stress in adhesives, the crack-stopping role of bolts, and stress concentration reduction [1,2]. Based on Mehrabian and Boukhili’s [1] experimental investigation on HBB joints, hybridization produces three positive effects: the reduction of secondary bending and twisting, the relief of stress concentration around the holes, and the delay of damage initiation, and thereby, the final fracture. They noted that the bolts prevented the free end debonding from progressing to the middle section. Gamdani et al. [2] experimentally investigated the tensile behavior of HBB joints and concluded that in three in-line bolt joint configurations, the exterior bolts controlled the peel stresses, whereas the adhesive reduced the stress concentration around the holes. Their study suggested the possible redundancy of the middle bolt and the effective transfer of load via the adhesive. Bodjona et al. [3] experimentally studied the effect of adhesive layer compliance on the strength of HBB joints. For a low-compliance adhesive, they observed no advantage in incorporating a fastener for a bonded joint, whereas it was noted that introducing a fastener failure for a high-compliance adhesive significantly postponed the onset of initial failure.

Kelly [4] studied various effects of load sharing in single-lap (SL) HBB joints using finite element analysis (FEA). They concluded that the load transferred by a bolt increases with increasing adherend and/or adhesive thickness and decreasing overlap length, bolt pitch distance, and/or adhesive modulus. In another study, Kelly [5] concluded that the benefits of using the HBB joint strength compared to bonded joints were only observed in joints with lower-modulus adhesives, enabling load sharing between the bolt and adhesive.

Based on the FEA model verified by experimental results, Bodjona and Lessard [6] reported that when the adhesive was not fully plasticized, the bolt did not transfer a significant portion of the applied load to the SL HBB joints. Moreover, reducing the bolt–hole clearance and regulating the adhesive thickness are effective methods for constraining the maximum plastic strain that develops in the adhesive along the overlapping edges. Armentani et al. [7] investigated an SL HBB composite joint using FEA. It was concluded that by reducing the strength of the structural adhesive, preload acting on the bolt, and the gap between the bolt shank and hole, the load on the bolt increased. Romanov et al. [8] conducted a parametric study on SL HBB composite joints under static tensile loading. They concluded that the major contributors to load sharing were the overlap length, bolt positioning, and flexible adhesives. Lopez-Cruz et al. [9] quantitatively evaluated the effect of several factors on the hybrid joint strength by applying the design of experimental methodology. They noted that hybridization offered superior performance compared to that of bonded joints because of its crack-arrest capability. Moreover, a higher joint strength can be achieved by decreasing the adhesive modulus, increasing the adherend thickness, and installing a bolt without clearance.

Some studies on HBB joints are currently available to compare FEA results with experimental results [10,11,12,13]. There are a few research works emphasizing the importance of calibrating FE results with experimental work. However, it is of crucial importance and needs to be performed before proceeding further with simulations. Additionally, the approach by which the woven fabric was modeled in FEA is one of the important aspects of this research. In the current study, the effect of hybridization on the strength of HBB joints and a comparison with respect to only bolted and bonded joints are conducted. Studies suggest that the layup significantly affects joint performance depending on its type [1], and therefore, two different layups are investigated: cross-ply (CP) and quasi-isotropic (QI).

## 2. Materials and Methods

### 2.1. Experimental Procedure

**Figure 1:**
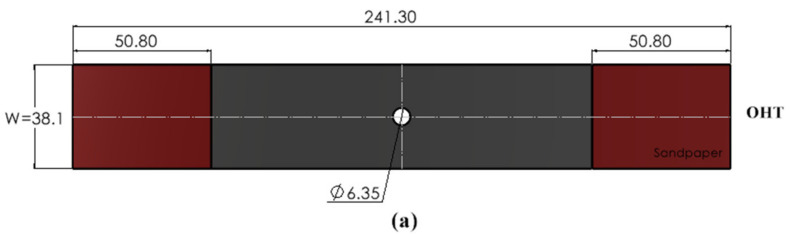

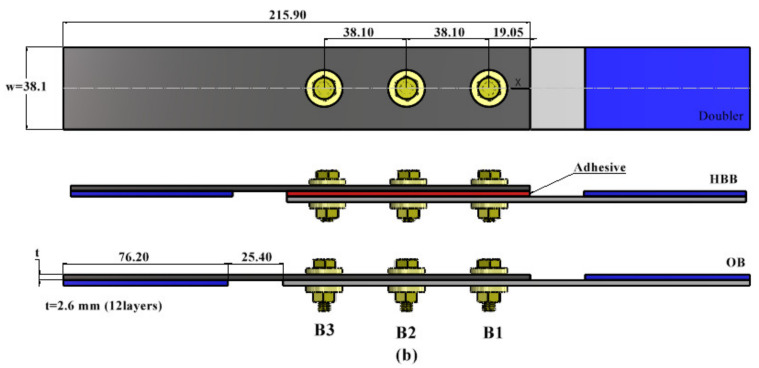
Specimen geometry and dimensions in mm for (**a**) OHT and (**b**) OB and HBB SL joints.

As stated previously, experimental tests were conducted to obtain the necessary input data for FEA modeling and to validate the simulation results. These experimental tests were performed at the Center for Applied Research on Polymers and Composites based on previous studies [1,14,15]. Three types of specimens, specifically, open-hole tension test (OHT), only bolted (OB), and HBB SL joints, were considered, as shown in Figure 1. It should be noted that the bolted joints contained three bolts, and the HBB or bonded joints had the same overlap length as the bolted joints.

The OHT was manufactured according to the ASTM D5766 standard [16], whereas SL shear bolted joints (OB and HBB) with three bolts were fabricated according to the ASTM-D5961 standard [17]. Hexagonal-head steel fasteners (NAS6204-4) with a shank diameter of 6.35 mm and nuts (MS21042-4) were used. These bolts were fitted with cadmium-plated steel washers (NAS1149F0463P) with an internal diameter of 6.73 mm and external diameter of 12.70 mm on both sides (head and nut sides), and an adhesive layer of 0.15 mm (Araldite^®^ LY 8601/Aradur^®^ 8602, Huntsman Corporation, Conroe, TX, USA) provided from Huntsman Corporation, Texas, USA was applied between the overlapping surfaces of the HBB joint.

The composite plates were made of carbon-fiber reinforced epoxy 3 K plain weave T300 carbon fabric and Araldite^®^ LY 8601/Aradur^®^ 8602 epoxy system using a vacuum-assisted resin transfer molding process [15]. All tensile tests of the OHT, OB, and HBB coupons serving as a baseline were conducted on a servo-hydraulic machine model 810 at an extension rate equivalent to 2 mm/min. A minimum of five coupons were used for each configuration to obtain a meaningful average of the experimental results. Table 1 lists the stacking sequence and fiber orientations of the CP and QI composites with 12 plies.

#### Digital Image Correlation Measurements

Digital image correlation (DIC) is an optical noncontact measurement technique that can determine the displacement fields in a loaded test specimen [18]. The full-field strain over the specimen surface was measured. This technique, coupled with the appropriate software, was used to determine the longitudinal strain fields of the laminates. The results of the three-dimensional digital image correlation (3D-DIC) used in this study are presented in detail in [1].

### 2.2. Numerical Simulation Procedure

The specimen geometries used to create the 3D simulation models of the OB, HBB, and OHT configurations are shown in Figure 1. An average laminate thickness of 2.6 mm for the 12 layers was measured on the experimental specimens.

#### 2.2.1. Theory Development

Classical laminate theory (CLT) [19] can be used to perform quick calculations to assess the behavior of a laminate and to calculate the stress and strain states in a ply within the laminate. The laminate stiffness matrix [ABD] was calculated considering the plane stress assumption, which was used to calculate the midplane strains with the applied loads. Equation (1) can then be used to determine the in-plane state of strains ($\varepsilon_{x},\;\varepsilon_{y},\;\gamma_{xy}$) for any specific ply *k* in the laminate as a function of its position $z_{k}$ along thickness direction.

(1) $${\left[\begin{array}{c} \varepsilon_{x}\\ \varepsilon_{y}\\ \gamma_{xy} \end{array}\right]}^{k}={\left[\begin{array}{c} \varepsilon_{x}^{0}\\ \varepsilon_{y}^{0}\\ \gamma_{xy}^{0} \end{array}\right]}^{k}+z_{k}{\left[\begin{array}{c} \kappa_{x}\\ \kappa_{y}\\ \kappa_{xy} \end{array}\right]}^{k}$$

Here, $\varepsilon_{x}^{0},\;\varepsilon_{y}^{0}$, and $\gamma_{xy}^{0}$ are the laminate mid-surface strains; $\kappa_{x}$ and $\kappa_{y}$ are the curvatures of the plate due to bending; and $\kappa_{xy}$ is the curvature of the plate due to twisting. Using the generalized Hooke’s law, the state of stress along the principal directions was determined.

#### 2.2.2. Materials Properties and Damage Modelling

**Figure 2:**
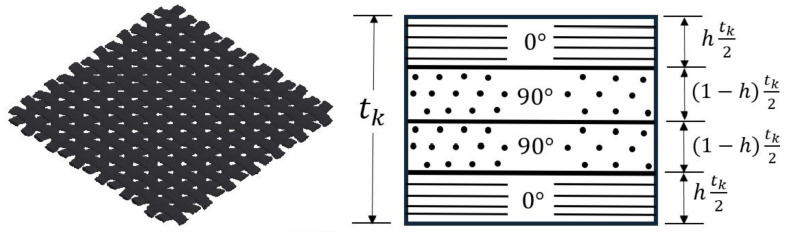
Woven ply simplification.

The fabrics were modeled using a technique discussed by Gordon et al. [20], which involves substituting one fabric ply with four unidirectional (UD) plies to form a symmetric sublaminate and stacking together to form the woven fabric laminate. This technique of replacing fabric layers with UD plies has been used by other researchers as well [21,22]. In addition, the sublaminate formed by the four UD plies has the same thickness as the fabric, but the thickness ratio of the ply complies with Equation (2), as suggested by [23].

(2) $$h=\frac{A_{w}}{A_{F}+A_{w}}$$

where $A_{w}$ is the warp fiber area, and $A_{F}$ is the fill fiber area, as shown in Figure 2. Because the fabric was plain, weave it had the same number of fibers in the fill and warp directions. Thus, h is equal to 0.5, and each UD ply has the same thickness, equivalent to 1/4 the thickness of the woven fabric ply. Thus, in the simulation, every woven ply was modeled by 4 UD plies.

The material properties used in the FEA model for the unidirectional plies are listed in Table 2. The out-of-plane properties were assumed to be the same as those in the transverse direction because they were driven by the matrix. $X_{t}$ and $X_{c}$ are the tensile and compressive strengths parallel to the fiber direction, respectively, $Y_{t}$ and $Y_{c}$ are the tensile and compressive strengths normal to the fiber direction, respectively, and $S_{L}$ and $S_{T}$ are the shear strengths along the fiber direction and normal to the fiber direction, respectively. $G_{Xt}$ and $G_{Xc}$ are the fiber fracture energies under tension and compression, respectively, and $G_{Yt}$ and $G_{Yc}$ are the matrix fracture energies under tension and compression, respectively. The fracture energies were obtained from the literature [24]. The results were then used to calculate the equivalent in-plane mechanical properties of the woven fabric considering the CLT.

**Figure 3:**
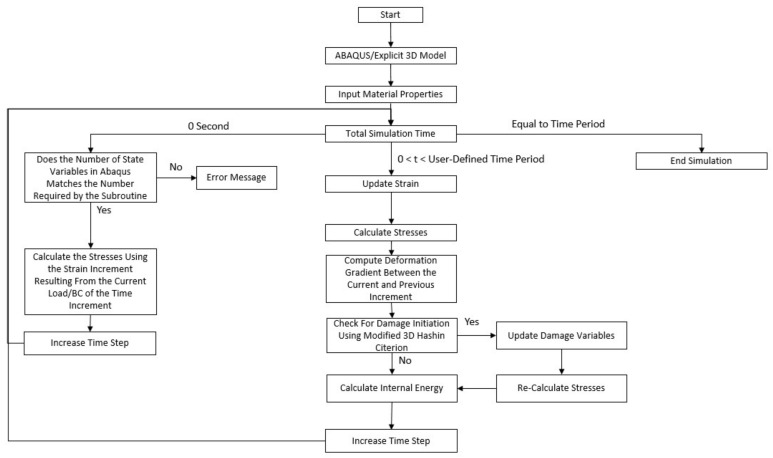
Damage initiation failure flow chart.

An improved Hashin failure criterion [25] was used to model the failure conditions of the individual laminae. The damage initiation criteria and damage evolution laws were programmed using a user-defined subroutine to predict four failure modes: tensile fiber damage initiation, compressive fiber damage initiation, tensile matrix damage initiation, and compressive matrix damage initiation. The corresponding subroutine flowchart is shown in Figure 3.

#### 2.2.3. Modelling of the Bolts and Washers

Bolts and washers made of steel were modeled as purely elastic isotropic materials. A Young’s modulus of 200 GPa, Poisson’s ratio of 0.3, and density of 8000 kg/m^3^ were used for this material. In the experiments, a bolt preload of 5 N.m was applied to the bolts [26]. By applying a temperature field to the washer, resulting in tension in the bolt, and considering a thermal expansion coefficient of 8.4 × 10^−5^ K^−1^, the temperature gradients for the simulation were obtained as listed in Table 3. A higher washer temperature was applied to the HBB joint configuration to achieve the desired preload because of the lower total out-of-plane stiffness of the assembly owing to the presence of the adhesive layer. The expansion coefficient was applied only along the thickness direction, whereas other orientations were insensitive to the applied temperature.

#### 2.2.4. Adhesive Properties

**Figure 4:**
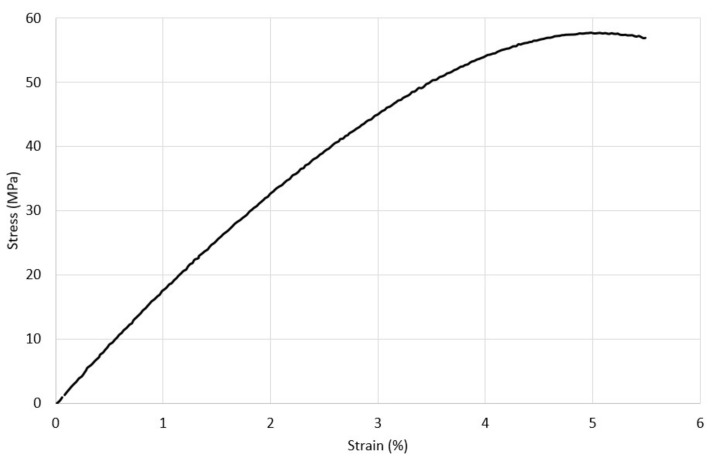
Araldite^®^ LY 8601/Aradur^®^ 8602 epoxy system uniaxial tensile stress–strain curve.

Araldite^®^ LY 8601/Aradur^®^ 8602 epoxy system adhesive is relatively rigid compared to those reported in [3,4]. Nevertheless, it was modeled as an isotropic elastoplastic material to account for any possible plastic deformation. The adhesive had a Young’s modulus of 1.61 GPa, Poisson’s ratio of 0.35, and density of 1090 kg/m^3^. The uniaxial tensile stress–strain curve of the adhesive is shown in Figure 4.

The cohesive properties of the adhesive are presented in Table 4 using data provided in references [27,28], including the shear strength of a comparable adhesive (EA 9628 Film Adhesive).

Here, $t_{n}^{0},\;t_{s}^{0},$ and $t_{t}^{0}$ are the ultimate normal and shear traction stresses, respectively; $k_{nn},\;k_{ss},\;and\;k_{tt}$ are the cohesive layer stiffness coefficients; and $G_{n}^{C},\;G_{s}^{C},\;G_{t}^{C}$ are the critical normal and shear fracture energies, respectively.

**Figure 5:**
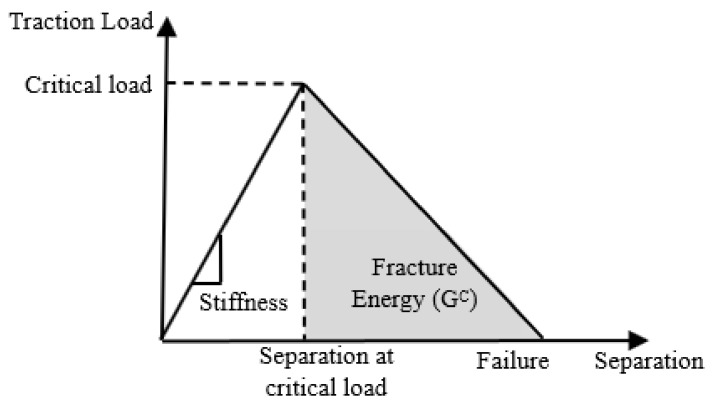
Linear traction separation cohesive zone model.

Abaqus contains a routine capable of implementing the cohesive zone model as a bilinear law comprising three main parameters: elastic stiffness, critical traction, and fracture energy, as schematically shown in Figure 5, representing the axial loading. A mixed-mode cohesive zone model was used for 3D modeling to predict the damage initiation using the quadratic nominal stress criterion [25]. The energy-based mixed-mode damage evolution law based on the fracture energy developed by Benzeggagh and Keane [29] was used to determine the critical fracture energy. It should be noted that the material parameter was assigned a value of 2, as suggested in [27] for a similar adhesive.

#### 2.2.5. Element Type and Mesh

To capture the high-stress gradients around the holes, the specimens were partitioned into regions that permitted smart meshing. First-order solid hexahedral reduced-integration elements were used throughout the model. A reduced integration scheme was used to reduce the computational cost and avoid shear locking, which is a common problem with fully integrated solid elements that causes an overly stiff model, leading to inaccurate results [30]. However, reduced integration elements can suffer from hourglassing because of a lower number of integration points. Thus, an enhanced hourglass-control algorithm was selected to artificially add stiffness to avoid hourglassing the element.

It was determined that the mesh refinement at the hole in the OHT model was sufficient for the other configurations (OB and HBB). For the HBB joints, a different partitioning method at the overlap ends was used to capture the stress gradient because the addition of an adhesive layer resulted in a high-stress concentration at the overlap ends.

#### 2.2.6. Contact Definitions and Mass Scaling

A general contact algorithm was used for the bolted joints. Each interaction was defined using a penalty friction model and a hard contact definition for the pressure-overclosure relationship to minimize the penetration of the surfaces in contact. The friction coefficients used were 0.7, 0.5, and 0.1 for laminate to laminate, bolt-to-washer, and bolt-to-washer-to-laminate interactions, respectively [31,32,33]. In the HBB models, general contact was used for all contact interactions. The only difference was in the contact between the adhesive layers, where a cohesive interaction was defined. No adhesive bondline failure was defined owing to the variability in the results, high dependency on surface preparation, and lack of experimental data. Therefore, the bondline was assumed to be perfect, and the adhesive layer was modeled as part of the laminate.

An extremely short time increment must be used to obtain a stable state. The stability limit is influenced by the minimum element characteristic length $L_{min}$ and dilatational wave speed in element $c_{d}$, which is proportional to the square root of the ratio between Young’s modulus and material density assigned to a specific element. Equation (3) was used in Abaqus to calculate the stable time increment of the most limiting element in the model [34].

(3) $$∆t=\min\left(\frac{L_{min}}{c_{d}}\right),\;c_{d}\propto \sqrt{\frac{E}{\rho }}$$

Because the simulation accuracy must not be decreased to accelerate the solution time, Young’s modulus must remain the same as that of the actual test specimen. Thus, the density is the only parameter that can be changed to accelerate the simulation. Instead of manually adjusting the density, Abaqus can scale the density of the critical elements such that the stable time increment increases.

## 3. Results

### 3.1. Material Model Calibration and Model Validation

The FEA models and experimental results were compared to confirm the accuracy of the simulation. For the validation process, the failure of the specimen/joint was defined as the peak load, after which the specimen/joint could not carry more load. Experimental results pertaining to plain-weave woven fabrics with various configurations were used to verify the ultimate strength and strain field around the holes.

#### 3.1.1. Experimental versus FEA Ultimate Failure Strength in OHT

The FEA-simulated tensile strength at the failure of the OHT specimens was compared with the experimental results for validation purposes. The results are presented in Table 5, and the agreement is very good. This confirms the validity of the material properties based on the methodology described in Section 2.2.2. Owing to the higher ply count in the loading direction, the CP layup had a higher strength, although this advantage was slightly attenuated by the fact that the CP layups were more notch-sensitive. However, independent of the layup, all the models failed because of tensile fiber failure.

#### 3.1.2. Longitudinal Strain Field in OHT

**Figure 6:**
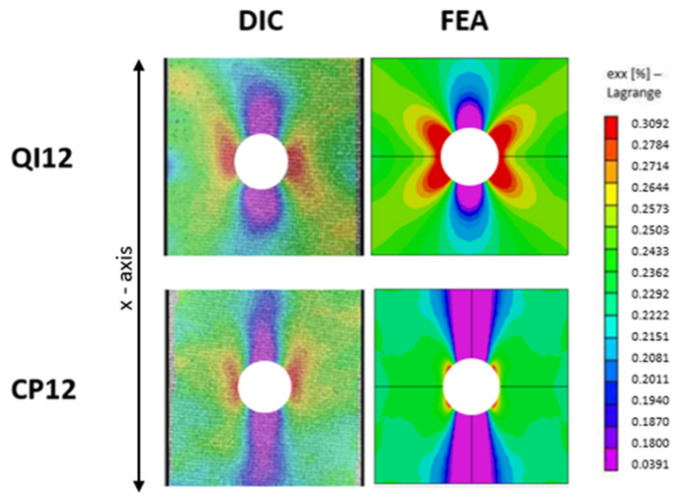
Strain field in the loading direction ϵ_xx_ at 25% of ultimate load for OHT CP12 and QI12 laminates comparing FEA with 3D-DIC results [14].

The longitudinal strain field results of the FEA and DIC experiments are shown in Figure 6. DIC presents a weakness in this case, as it does not allow for the proper measurement of the strain at the edge of the hole. The FEA results were adjusted to reflect the state of uncaptured regions.

Reasonable agreement was revealed between the simulation and experiment. As expected, a high-strain concentration region was found at the hole edge, indicating the stress concentration generated by the discontinuity. Similar strain levels for the same stress level indicate an accurate estimation of the stiffness properties of the FEA models. The high-strain regions originating from the hole edge tended to stretch along the loading direction (0° layers) for the CP layup and in the 45° direction for the QI layup. The same trend observed for the QI layup was reported for isotropic materials such as aluminum [35]. The increased notch sensitivity of the CP layups compared to the QI layups reported in [1] is depicted as strains that reduce quickly when moving away from the hole in the CP layups.

#### 3.1.3. Nominal Stress–Displacement Curves in OB and HBB Joints

**Figure 7:**
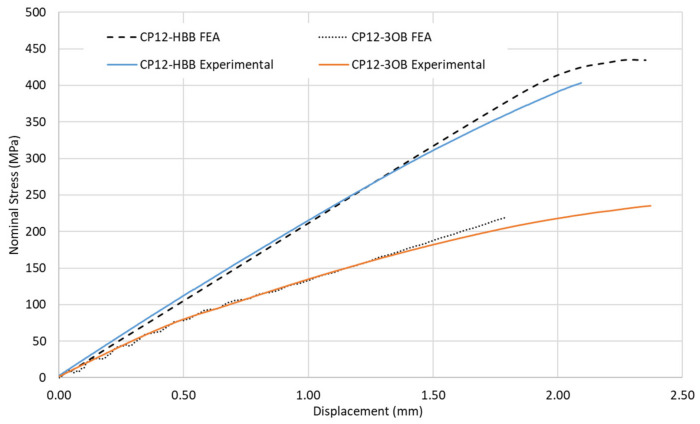
Nominal stress–displacement curves for OB and HBB Joints, case of CP layup.

**Figure 8:**
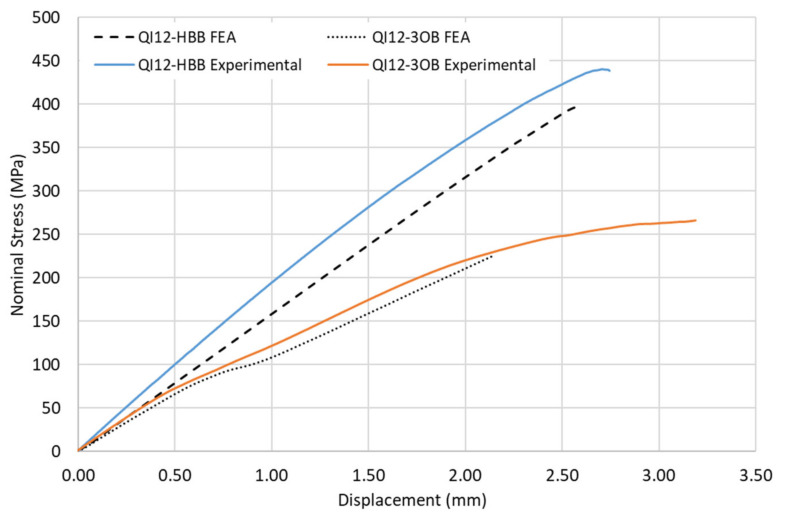
Nominal stress–displacement curves for OB and HBB Joints, case of QI layup.

A comparison between the experimental and simulation results considering the bolted and hybrid joints for the CP and QI layups is shown in Figure 7 and Figure 8. Improvement in the performance of the HBB joint is noticeable. A good agreement between the predicted and actual results was observed for the CP case. However, a weaker correlation was noted for the QI layup of the HBB joint. It was noted during the experiment that the QI laminates had a slightly higher fiber volume fraction content. However, in the simulation, the same mechanical properties were used independently of the layup, which creates this discrepancy. Additionally, more conservative results were obtained for the OB joint caused by the sensitivity of Hashin failure criteria to the high shear stresses developing at the loaded holes.

#### 3.1.4. Longitudinal Strain Field in OB and HBB Joints

**Figure 9:**
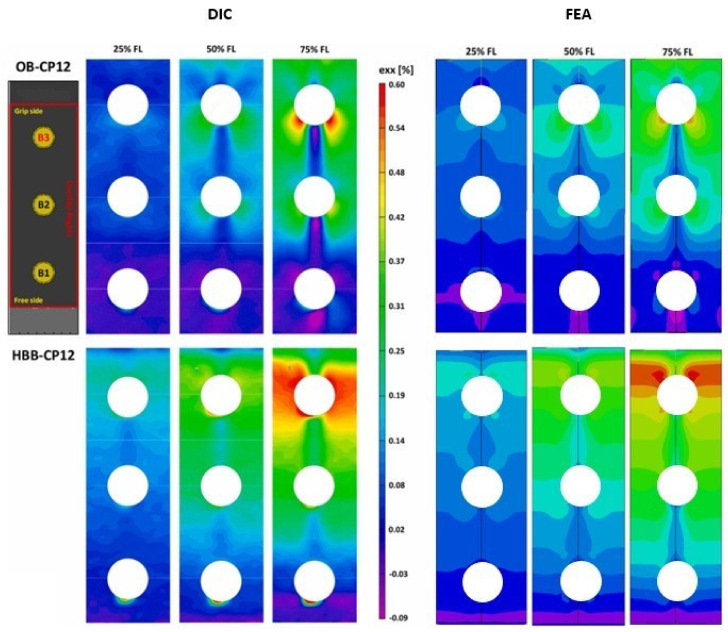
Longitudinal strain field for the CPlayup away from the washer; comparison between 3D-DIC results [1] and simulation.

**Figure 10:**
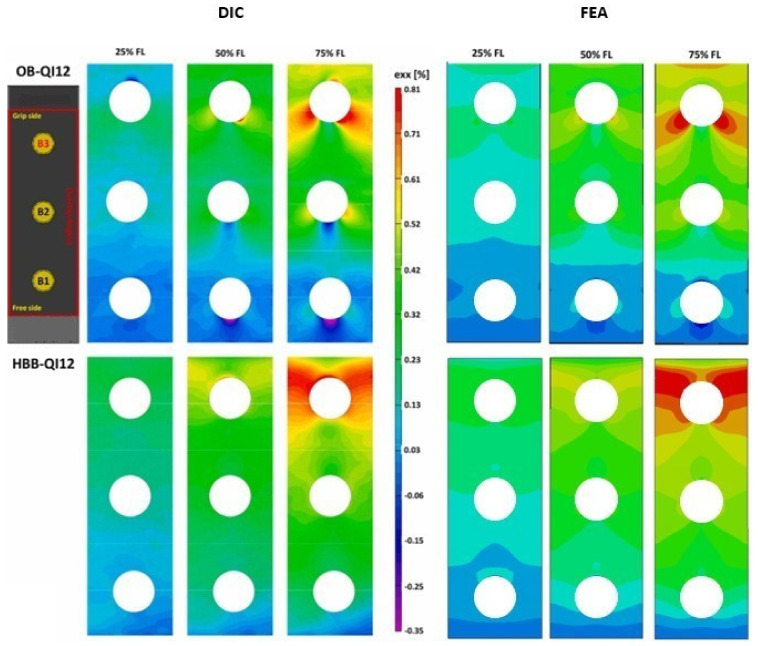
Longitudinal strain field for the QI layup away from the washer; comparison between 3D-DIC results [1] and simulation.

A comparative assessment of the strains acting along the loading axis and at the external surface of the 0° layer was conducted. For joint configurations, DIC presents an inherent limitation because the washers prevent strain measurements on the laminate near the holes. Therefore, only the region away from the washer was used to validate the FEA model. These results are depicted in Figure 9 and Figure 10 with consideration to the fact that they are presented as a percentage of their corresponding failure load.

The effects of the bolts on the OB and stress concentration reduction at the holes caused by the addition of the adhesive in the HBB joints were noticeable and adequately captured by the simulation. The bolt load distribution phenomenon, which commonly occurs in multiple fastener joints, develops a strain gradient originating at the beginning of the overlap and propagating towards the grip end. This trend was independent of the layup type. In the case of the HBB, the analysis of the strain field pattern in the QI layups revealed a reduction in the effects generated by the 45° layers, resulting in a similar behavior to that of the CP layups. Higher longitudinal strains were observed in the QI layups owing to their lower stiffness. However, regardless of the layup, the high-strain region propagated at a 45° angle at the hole in the OB joints, owing to the bearing pressure applied by the bolt shank. Thus, it is observed that while the type of layup influences the strain levels, the joining method has the most significant influence on the strain field pattern.

A comparison of the FE load–displacement behavior, ultimate strength, and longitudinal strain field for different tensile test configurations (OHT, OB, and HBB joints) with experimental data proved that the modeling strategy could yield accurate results. This is highly pertinent because high-fidelity results can be obtained at a relatively low computational cost using FEA while using a woven fabric.

### 3.2. Assessment of The Behavior for Different Joining Methods

**Figure 11:**
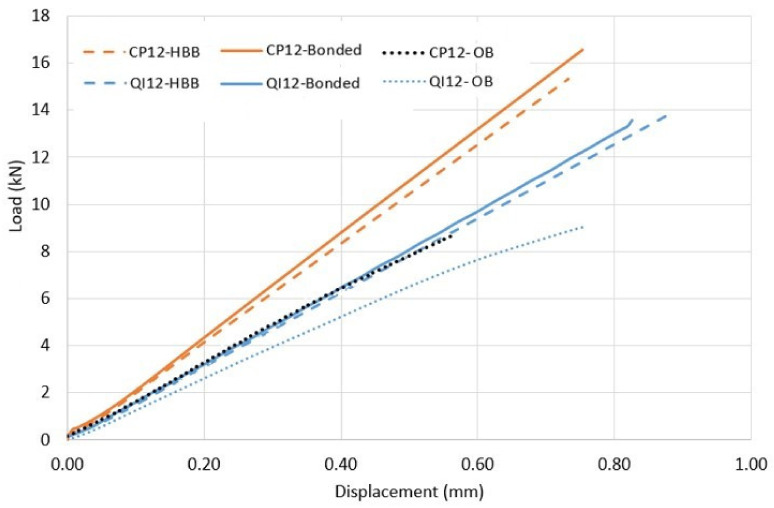
Load–displacement curves for QI12 and CP12 layups.

The load–displacement relationship, as predicted by the simulation, is shown in Figure 11 to provide a comparison of the behavior of bolted, bonded, and hybrid bonded-bolted joints subjected to the same loading condition until failure for each case layup. It should be noted that failure is defined as the onset of fiber and/or adhesive damage. Table 6 summarizes the observed failure modes and their corresponding failure loads.

These results confirm the benefits of the adhesive in comparison to those of the fasteners, intensified by the favorable alignment of most layers in the case of the CP configuration. For OB joints, the QI configuration presents a slight advantage in strength owing to the presence of 45° plies, which increases the joint capability of sustaining bearing loads. Additionally, the bonded and HBB QI joints have lower strengths owing to higher peel and shear stresses compared to the CP layups. In addition, it appears that the behavior and strength of the HBB joint are similar to those of a bonded joint, suggesting that the adhesive is the main contributor to the load transfer between the laminates.

**Figure 12:**
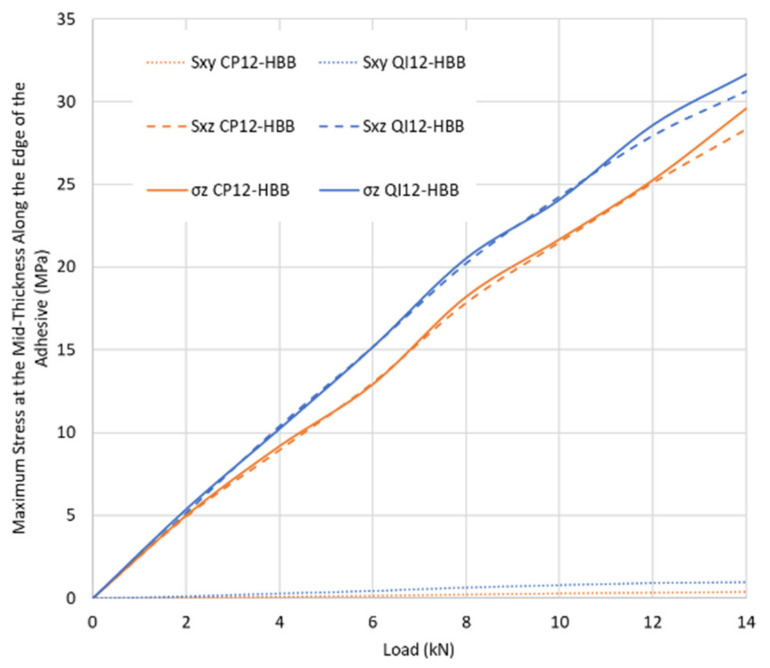
Comparison of stresses in the adhesive layer for QI12 and CP12 HBB joints.

**Figure 13:**
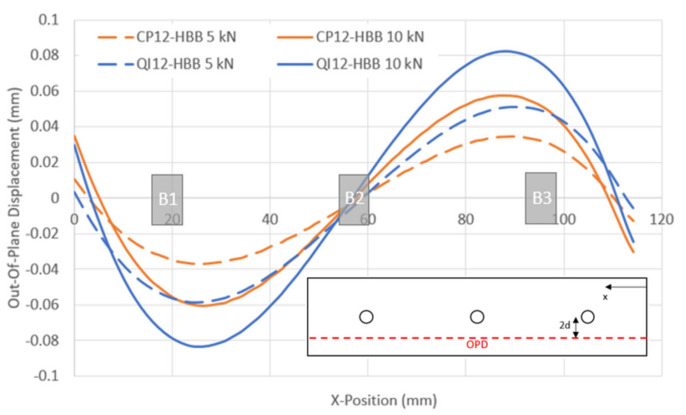
Comparison of the out-of-plane displacement for QI12 and CP12 HBB joints.

The layup was found to influence the stress levels in the adhesive, as shown in Figure 12. For the same load level, the adhesive in the QI-HBB joint will be more stressed in peel (σ_z_) and shear (S_xz_) than in the CP-HBB joint. This is why CP-bonded joints performed better than QI-bonded joints. The difference in peel stresses can be attributed to the fact that CP layups are much stiffer than QI layups due to the higher 0° ply count. Since a correlation exists between longitudinal stiffness and bending stiffness, the same comparison can be made regarding the superior bending stiffness of CP layups, meaning less out-of-plane displacement is observed for a joint using a CP layup, as shown in Figure 13. It is also further emphasized that the main contributors to adhesive damage are S_xz_ and σ_z_ since the in-plane shear stress S_xy_ is so low that it is an insignificant component of the adhesive failure.

#### 3.2.1. Out-Of-Plane Displacement for Different Joining Methods

**Figure 14:**
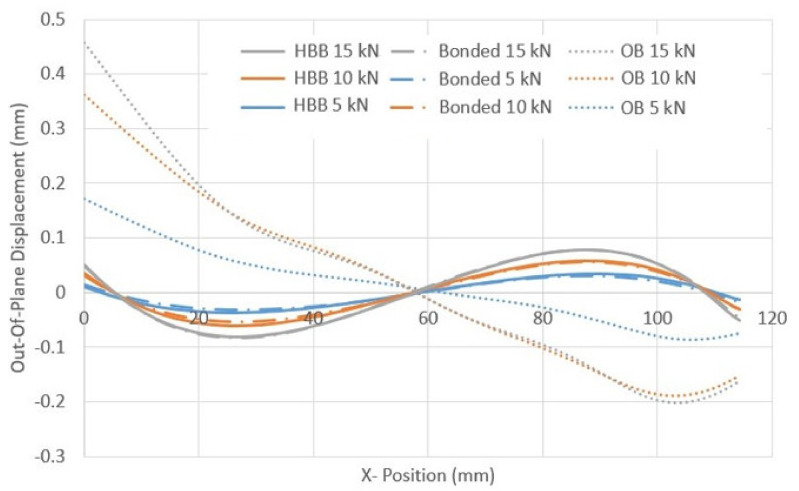
OPD for different joining methods using CP12 at various load levels.

The out-of-plane displacement (OPD) is shown for different joint configurations at different load levels in order to see the effect of adhesive behavior in Figure 14. The results suggest that the adhesive mainly drives the hybrid joint behavior because the HBB and bonded joints have the same OPD curves for every load level. Additionally, compared to OB, HBB joints suffer from significantly less OPD, and the behavior of the joint over the overlap length is very different. In the HBB joint, the adhesive layer maintains the two laminates together, even at the overlapping ends, which not only reduces the maximum/minimum OPD by up to 84% with respect to the OB joint, as shown in Table 7, but also gives a sinusoidal trend to the out-of-plane behavior of the joint. Thus, the HBB joint retained its original geometry better than the OB joints in a structure, even at higher load levels.

**Figure 15:**
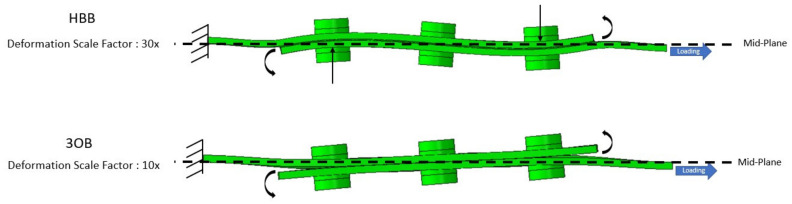
Scaled OPD of OB and HBB joints at 8 KN, case of CP12 layup.

The difference in the OPD behavior between the OB and HBB joints was mainly due to the presence of the adhesive layer. Figure 15 shows the joint deformation under tensile-shear loading. The scaling increased for the HBB joint configuration as it displayed significantly less OPD than the OB joint. Owing to the eccentricity in the load path, secondary bending occurred in both joints in an attempt to separate the laminates at the overlapping ends. In the OB joints, this rotation at the overlapping ends is unrestrained, and the joint opens, resulting in a large OPD at the overlapping end. In the HBB joints, the adhesive layer resists rotation at the overlapping ends, consequently inducing a sinusoidal form in the OPD owing to the restriction of the joint opening at the overlapping ends. Moreover, because both ends resist secondary bending in the HBB, the OPD in the middle of the overlap is zero.

#### 3.2.2. Longitudinal Strain Field Comparison

The main objective of the strain-field analysis presented thus far was to validate the simulation model against the available experimental results. Only a few conclusions on the behavior of the joints when comparing OB with HBB were possible because the experimental data obtained using DIC were collected at loading-level functions of the ultimate resistance of the joint. In addition, the DIC technique does not allow measurements under the washer; therefore, the strain fields closer to the holes in the critical zones of concentrated stress cannot be captured.

**Figure 16:**
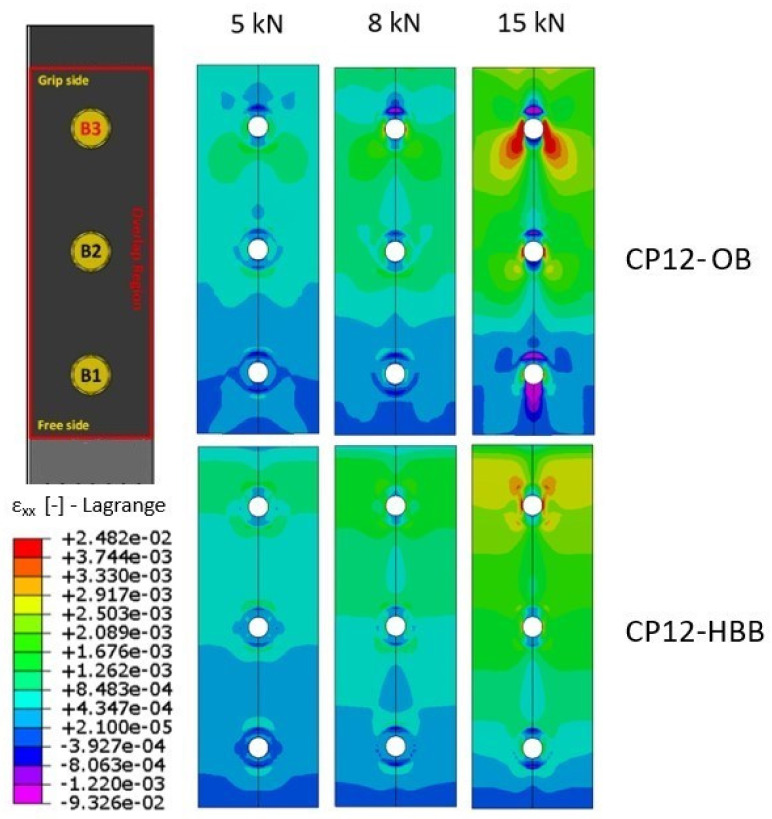
Longitudinal strain field comparison using simulation depending on the joint configuration, case of CP layup.

**Figure 17:**
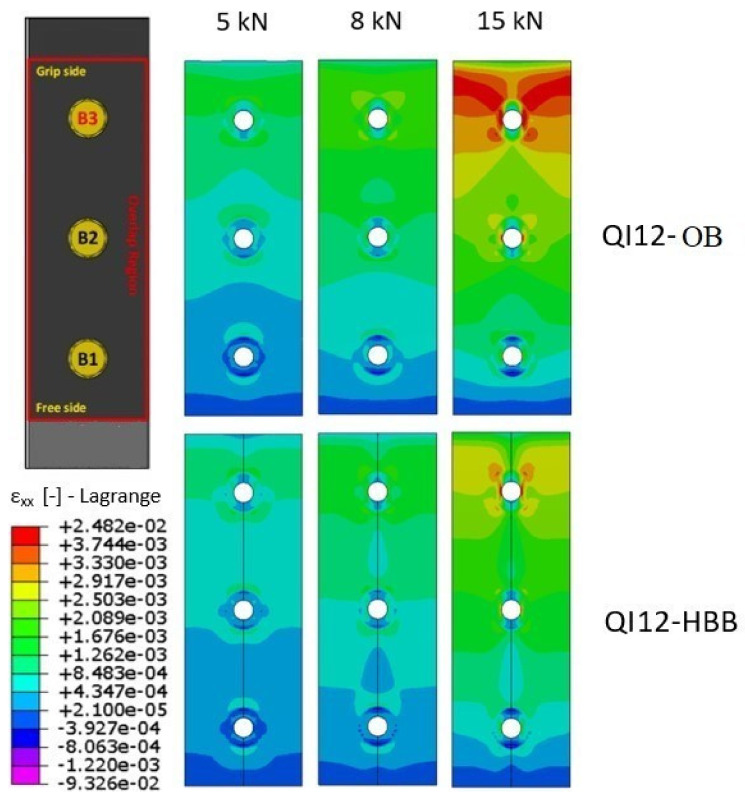
Longitudinal strain field comparison using simulation depending on the joint configuration, case of QI layup.

Based on the simulation results, Figure 16 and Figure 17 show the strain fields for the OB and HBB joint configurations at various load levels. The analysis of the strain field around the most loaded region of the laminate reveals a net advantage of the increased strength of the HBB yielded by the addition of the augmented adhesive, especially for CP layups, owing to the favorable orientation of a higher number of plies with respect to the loading direction. These results suggest a significant strain concentration relief at all holes owing to hybridization, especially at the critical hole. It should be noted that independent of the joint configuration and layup, the critical region where high deformations occur is at the hole corresponding to B3. This is because this hole is situated closer to the grip side, and thus, significantly higher bypass loads are observed at this position, contributing to the development of higher strains.

## 4. Discussion and Conclusions

In summary, a finite element investigation of the mechanics of hybrid bolted/bonded joints based on experimental tests was conducted by analyzing the stresses in the critical regions of the joint. To achieve this, several critical aspects regarding the modeling of plane-weave material properties, adhesive properties, and mass-scaling definition must be considered. To perform material calibration, the stress and strain results based on FEA were compared with the experimental results for the open-hole tension test, three OB as well and HBB joints to ensure the validity of the results.

Based on the FEA results, the HBB joints could carry significantly more load than the OB joints when the first significant failure event was considered. Moreover, the OPD of the HBB joints decreased considerably with respect to that of the OB joints owing to the secondary bending resistance of the adhesive at the overlapping ends. However, the maximum strength potential of the HBB joint was limited to the bonded joint strength because the stresses in the adhesive layer were the same for the bonded and HBB joints. CP layups generally depict higher strength than QI layups due to the existence of more aligned fibers in the tensile direction, resulting in higher load failure and lower longitudinal strain magnitudes. Moreover, the stresses were localized around the hole closer to the grip side in the laminates and spread at an angle of approximately 45° within the OB joints, owing to the bearing pressure exerted by the bolt shank. In contrast, for the HBB joints, the strain field exhibited a more uniform shape and was significantly reduced.

The FE study did not consider cracks initiating from flaws before high stresses developed in the adhesive. This limitation prevents any conclusion as to whether damage initiation or propagation in the adhesive is more detrimental to the joint’s performance. Additionally, the impact is a regime of concern with composite laminates. Even low-energy impacts can lead to barely visible damage in laminates. Thus, an impact analysis of HBB joints would be interesting to capture the effect of sudden damage initiation on the integrity of the joint configuration under such loading conditions.

## Figures and Tables

**Table 1:** Stacking sequence of studied specimens.

CP Symmetric Sequence-12 Plies (CP12)	QI Symmetric Sequence-12 Plies (QI12)
[(0/90)/(0/90)/(0/90)/(0/90)/(0/90)/(0/90)]_S_	[(0/90)/(±45)/(0/90)/(±45)/(0/90)/(±45)]_S_

**Table 2:** FEA unidirectional laminae properties.

Property	Value
$t_{ply}$	0.055 mm
Density	1460 kg/m^3^
Elastic properties	$E_{1}=100\;GPa,{\;E}_{2}=E_{3}=7\;GPa$
	$G_{12}=G_{13}=G_{23}=4\;GPa$
	$\nu_{12}=\nu_{13}=0.3$, $\nu_{23}=0.35$
Strength	$X_{t}=1150\;\mathrm{MPa},\;X_{c}=1000\;\mathrm{MPa}$
	$Y_{t}=54\;\mathrm{MPa},\;Y_{c}=250\;\mathrm{MPa}$
	$S_{L}=S_{T}=110\;\mathrm{MPa}$
Fracture toughness [24]	$G_{Xt}=91.6\frac{Jm}{{\mathrm{mm}}^{2}},\;G_{Xc}=79.9\frac{Jm}{{\mathrm{mm}}^{2}}$
	$G_{Yt}=0.22\frac{Jm}{{\mathrm{mm}}^{2}},\;G_{Yc}=1.1\frac{Jm}{{\mathrm{mm}}^{2}}$

**Table 3:** Temperature gradients used in the simulation process of the clamping load.

Joint Type	ΔT (K)
OB	147
HBB	188

**Table 4:** Cohesive surface properties.

$t_{n}^{0}$ (MPa)	$t_{s}^{0}$ (MPa)	$t_{t}^{0}$ (MPa)	$k_{nn}\;(N/mm^{3})$	$k_{ss}\;(N/mm^{3})$	$k_{tt}\;(N/mm^{3})$	$G_{n}^{C}$ $\left(mJ/{mm}^{2}\right)$	$G_{s}^{C}$ $\left(mJ/{mm}^{2}\right)$	$G_{t}^{C}$ $\left(mJ/{mm}^{2}\right)$
55	39.3	39.3	$10^{5}$	${3.575\times 10}^{4}$	${3.575\times 10}^{4}$	2.5	5	5

**Table 5:** OHT ultimate failure strength.

Parameter	CP12	QI12
OHT FEA Failure Stress (MPa)	423	364
OHT Experimental Failure Stress (MPa)	409	366
Discrepancy (%)	3.2	−0.5

**Table 6:** Failure load of different joint configurations.

Model	Failure Mode	Failure Load (KN)
HBB Joint (CP12)	Fiber Failure	15.41
OB Joint (CP12)	Fiber Failure	8.72
Bonded Joint (CP12)	Adhesive Failure	16.50
HBB Joint (QI12)	Adhesive Failure	13.78
OB Joint (QI12)	Fiber Failure	9.01
Bonded Joint (QI12)	Adhesive Failure	13.58

**Table 7:** Comparison of OPD between OB and HBB Joints (CP12).

Magnitude of Tensile Load	Maximum OPD Value for OB Joint (mm)	Maximum OPD Value for HBB Joint (mm)	Difference in Maximum OPD (%)
5 KN	0.171	0.035	79.8
10 KN	0.362	0.057	84.1
15 KN	0.458	0.079	82.7

## Data Availability

No data were used in the study described in this article.
